# Sleep-related breathing disorder in a Japanese occupational population and its association with hypertension—stratified analysis by obesity status

**DOI:** 10.1038/s41440-024-01612-y

**Published:** 2024-03-04

**Authors:** Minako Inoue, Satoko Sakata, Hisatomi Arima, Ikumi Yamato, Emi Oishi, Ai Ibaraki, Kenichi Goto, Takanari Kitazono

**Affiliations:** 1 Department of Medicine and Clinical Science, Graduate School of Medical Sciences, Kyushu University, Fukuoka, Japan; 2 Center for Cohort Studies, Graduate School of Medical Sciences, Kyushu University, Fukuoka, Japan; 3 Department of Epidemiology and Public Health, Graduate School of Medical Sciences, Kyushu University, Fukuoka, Japan; 4 Department of Preventive Medicine and Public Health, Fukuoka University, Fukuoka, Japan; 5 Department of Health Sciences, Graduate School of Medical Sciences, Kyushu University, Fukuoka, Japan

**Keywords:** Sleep-related breathing disorder, Hypertension, Obesity

## Abstract

Sleep-related breathing disorder (SRBD) causes hypertension, and obesity has been highly associated with SRBD, which has become a serious health problem in young and middle-aged Japanese males. However, the relation between SRBD and hypertension considering the effects of obesity remains unknown. In this cross-sectional study, we examined the relationship between SRBD and hypertension, with consideration for the effects of obesity, in Japanese occupational population. Using 3% oxygen desaturation index (3%ODI) obtained by simplified polysomnography (PSG), participants were classified into low (0 ≤ 3%ODI < 5), medium (5 ≤ 3%ODI < 15), and high (15 ≤ 3%ODI) 3%ODI groups. We excluded employees who had not undergone medical examination with simplified PSG in the same year from 2012 to 2018. Logistic regression analysis was performed to calculate odds ratios for having hypertension according to 3%ODI levels. In total, 2532 employees were included. Among them, 25% and 4% were categorized into the medium and high 3%ODI groups, respectively. The odds ratio for hypertension increased significantly with higher 3%ODI levels after adjustment for age, sex, alcohol drinking status and smoking status (*p* for trend < 0.0001). However, further adjustment for obesity status (body mass index ≥ 25 kg/m^2^) attenuated the associations. When we performed the stratified analysis by obesity status, the odds ratio for hypertension increased significantly with higher 3%ODI only for non-obese individuals, with significant interaction (p for interaction = 0.014). Higher 3%ODI was significantly associated with higher prevalence of hypertension especially in non-obese participants, suggesting the importance of vigilance for the presence of SRBD even in non-obese individuals.

**Figure:**
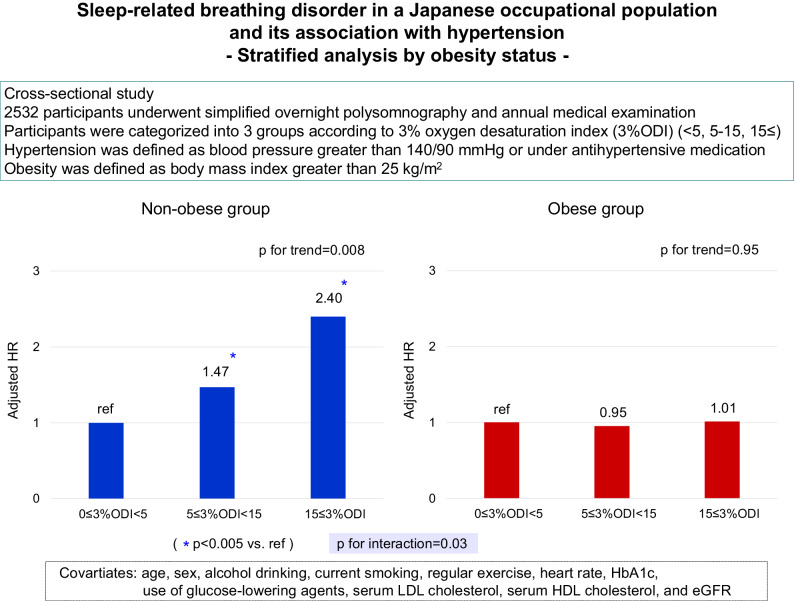
We investigated the association between SRBD and hypertension considering the effects of obesity, which would suggest the need to keep in mind the presence of SRBD even in non-obese individuals.

We investigated the association between SRBD and hypertension considering the effects of obesity, which would suggest the need to keep in mind the presence of SRBD even in non-obese individuals.

## Abstract (web summary)

We investigated the association between SRBD and hypertension considering the effects of obesity, which would suggest the need to keep in mind the presence of SRBD even in non-obese individuals.

## Introduction

Sleep-related breathing disorder (SRBD) and hypertension are highly comorbid, and SRBD itself causes hypertension [1–5]. Past epidemiological studies suggest that obstructive sleep apnea with apnea hypopnea index (AHI) is 5 or more account for most cases of SRBD. AHI represents the total number of apnea and hypopnea incidents per hour during sleep.

SRBD can contribute to hypertension, independently of older age and obesity [6–8]. The Wisconsin Sleep Cohort study, a prospective study in a regional general population, showed that increased AHI was associated with the increased risk of developing hypertension independently of older age and higher body mass index (BMI) [7]. The Sleep Heart Health Study showed that the impact of SRBD on the development of hypertension was greater in younger people and decreased in older people [8]. In Japan, the prevalence of hypertension in young and middle-aged men has been slightly increased, especially in hypertensive patients with obesity (JSH 2019). SRBD could be the cause of this trend, although further studies will be needed to confirm this. In the Japanese general population, it has been reported that SRBD is associated with hypertension [9], and that obesity is associated with hypertension, especially in younger generation [10]. However, the very few have examined the association between SRBD and hypertension with consideration for the effects of obesity status. In this study, we investigated the association between SRBD and hypertension with consideration for the effects of obesity, in a Japanese occupational population.

Point of view

- Clinical relevance
  The presence of SRBD should be considered even in non-obese individuals.
- Future direction
  A prospective study to investigate the causal relationship between 3%ODI elevation and blood pressure elevation is warranted.
- Consideration for the Asian population
  Asians tend to have small jaws, and the possible presence of SRBD and its subsequent effect on blood pressure should be considered even in non-obese individuals.

## Methods

### Study population

The cross-sectional study enrolled employees of a railroad and bus company based in Fukuoka, Japan. Study participants were enrolled between 2012 and 2018, who underwent both simplified overnight polysomnography (PSG) and annual medical examination in the same year. Simplified PSG was conducted once every 3 years for active train and bus drivers who were judged fit for driving with passengers. Of those, 105 drivers who were already on Continuous Positive Airway Pressure (CPAP) treatment were excluded. Finally, 2532 participants were enrolled in the present study. When the participants underwent the simplified PSG more than once during the period, the first results were used in this analysis.

The research proposal for this study was prepared in accordance with the “Ethical Guidelines for Medical and Health Research Involving Human Subjects.” Study approval was obtained from the Ethical Review Committee of the Kyushu University Medical School and Hospital. We explained the use of the data for this study at the time of the annual medical examination and posted the content and purpose of this study on the website of the Department of Medicine and Clinical Science, Graduate School of Medical Sciences, Kyushu University. Employees were excluded if they declined the use of their data for this analysis.

### Measurement of blood pressure levels and confounding variables

Physical measurements, administration of lifestyle questionnaires, and blood and urine tests were conducted at the annual health examination. Height, weight, sitting blood pressure, and pulse were measured, and BMI was calculated as weight (kg)/height (m) squared. Obesity was defined as BMI greater than 25 kg/m^2^ [11]. Blood pressure values in the sitting position were measured by a mercury sphygmomanometer in 2012 and 2013, and by an electronic oscillometric sphygmomanometer (ES-H55, Terumo Inc., Tokyo, Japan) from 2014 to 2018. The measurements were repeated and the steady values of blood pressure were used for the present analysis. Hypertension was defined as blood pressure greater than 140/90 mmHg and/or under antihypertensive medication [12]. Prehypertension was defined as blood pressure greater than 130/80 mmHg and less than 140/90 mmHg without the use of antihypertensive agents [12]. Heart rate was measured by resting electrocardiogram. Blood sampling was performed regardless of before or after meals. (Incidentally, frequency of fasting blood sampling was higher.) Diabetes mellitus was defined as hemoglobin A1c (HbA1c) greater than 6.5% and/or current use of glucose-lowering agents (i.e., oral glucose-lowering agents or insulin) [13]. Blood glucose levels were not used for definition of diabetes mellitus. Dyslipidemia was defined as serum low-density lipoprotein (LDL) cholesterol greater than 140 mg/dL and/or serum high-density lipoprotein (HDL) cholesterol less than 40 mg/dL and/or current use of lipid-modifying agents [14]. Triglycerides were not used for definition of dyslipidemia. Estimated glomerular filtration rate (eGFR) was calculated using the Chronic Kidney Disease Epidemiology Collaboration equation with a Japanese coefficient of 0.813 [15]. Regarding lifestyle, the participants were asked to fill out questionnaires on drinking, smoking, and exercise in advance, and the nurses additionally asked about any missing information or details on the day of the medical checkup. Drinking was defined as drinking at least once a week, regardless of the amount of alcohol consumed. Alcohol consumption was expressed as weekly ethanol consumption. Smoking was defined as current smoking, regardless of the number of cigarettes smoked. The number of cigarettes smoked was expressed as Brinkman index (number of cigarettes smoked per day multiplied by number of years smoked), regardless of whether or not the person is currently smoking. Regular exercise was defined as exercising at least twice a week.

### Assessment of sleep-related breathing disorder

In this study, SRBD was assessed using a simplified overnight SpO2 monitor with memory rather than full PSG. The validity of the simplified PSG has been confirmed in several studies [16–19]. Either of two simplified PSG devices was used. The PMP-200GpluxX (Royal Phillips Co., Amsterdam, Netherlands) or PULSOX-Me300 (Minolta Co., Osaka, Japan). Participants performed the test at home, as instructed by nurses. After attaching a pad to the wrist of the non-dominant arm, the sensor probe was attached to the index finger and secured with the provided tape. The power button was held down for at least 2 s, and the monitor on the pad confirmed that the measurement had started. The collected data were analyzed by Philips collectively. SRBD was defined according to the International Classification of Sleep Disorder, 3rd Edition (ICSD-3), with 3%ODI 5 events/h or more as mild, and 3%ODI ≥ 15 events/h as moderate or severe [20].

### Statistical analysis

The distributions of variables were evaluated by visually checking the histograms and by performing normality test using Kolmogorov-Smirnov test and Anderson-Darling test. Consequently, the distributions of ethanol consumption and Brinkman index were skewed. The trends in the means (standard deviations) and the frequencies of risk factors across the levels of 3%ODI were estimated by a linear and a logistic regression analysis, respectively. The trends in the medians (interquartile ranges) across the levels of 3%ODI were estimated by a nonparametric method (Wilcoxon rank sum test). Adjusted blood pressures (standard errors) and adjusted odds ratios (95% confidence intervals) for having hypertension according to 3%ODI levels were estimated by using analysis of covariance and logistic regression models, respectively. We used three models in the analysis: model 1 was adjusted for age and sex; model 2 was adjusted for the variables included in model 1, current alcohol drinking, current smoking, regular exercise, heart rate, HbA1c, use of glucose-lowering agents, serum LDL cholesterol, serum HDL cholesterol, and eGFR; and model 3 was adjusted for the variables included in model 2 and obesity status. Adjustment factors were potential confounding factors selected from the background of the participants and referred to adjustment factors in the similar previous studies [9, 21]. Multi-nominal logistic regression was also performed to evaluate the odds ratios for having prehypertension and hypertension according to 3%ODI levels. All statistical analyses were performed using the SAS program package version 9.4 (SAS Institute, Cary, NC). *P* < 0.05 were considered significant.

## Results

The mean age of the participants was 43 ± 11 years (interquartile range 37–51 years), 96% were male (2433 males and 99 females), and 10% (259 participants) were taking antihypertensive medication.

The median 3%ODI was 2.9 events/h (interquartile range 1.3–5.6). The frequencies of mild SRBD (5 ≤ 3%ODI < 15) and moderate or higher SRBD (3%ODI 15 or higher) were 25% and 4%, respectively. Table 1 shows the baseline characteristics of the total study population and the mean values or frequencies of potential risk factors for hypertension according to the 3%ODI levels. The mean values of age, BMI, systolic and diastolic blood pressure, HbA1c, serum total cholesterol, serum LDL cholesterol and serum HDL cholesterol increased significantly with higher 3%ODI levels. The mean values of eGFR decreased significantly with higher 3%ODI levels. The frequencies of obesity, current alcohol intake, lipid-modifying agent use, dyslipidemia, antihypertensive medication and hypertension increased significantly with higher 3%ODI levels.

**Table 1:** Baseline characteristics of the total study population

	3% oxygen desaturation index			
	0 ≤ 3%ODI < 5	5 ≤ 3%ODI < 15	15 ≤ 3%ODI	*p* for trend
	(*n* = 1804)	(*n* = 627)	(*n* = 101)	
Age (years)	42 ± 11	46 ± 10	46 ± 9	<0.0001
Sex (men, %)	95.4	98.1	96.0	0.02
BMI (kg/m^2^)	22.9 ± 3.0	24.8 ± 3.3	26.4 ± 3.8	<0.0001
Obesity (BMI ≥ 25 kg/m^2^, %)	21.5	42.9	63.4	<0.0001
Current alcohol intake (%)	54.6	60.8	68.3	0.002
Ethanol consumption (g/week)	5.0 (0.0–21.0)	12.0 (0.0–28.0)	20.0 (0.0–35.0)	<0.0001
Current smoking (%)	45.5	45.9	37.6	0.28
Brinkman index	240 (0–480)	315 (48–560)	315 (0–540)	<0.0001
Regular exercise (%)	15.8	16.0	11.9	0.56
Systolic blood pressure (mmHg)	119 ± 12	123 ± 13	124 ± 10	<0.0001
Diastolic blood pressure (mmHg)	76 ± 10	79 ± 10	81 ± 9	<0.0001
Heart rate (bpm)	71 ± 12	72 ± 11	75 ± 12	<0.0001
Hypertension (%)	16.3	26.6	33.7	<0.0001
Antihypertension medication (%)	8.2	14.2	22.8	<0.0001
HbA1c (%)	5.6 ± 0.5	5.7 ± 0.6	5.8 ± 0.6	<0.0001
Glucose-lowering agent use (%)	2.1	2.9	2.0	0.49
Diabetes Mellitus (%)	4.6	6.9	6.9	0.06
Serum total cholesterol (mg/dL)	202 ± 34	211 ± 36	214 ± 38	<0.0001
Serum LDL cholesterol (mg/dL)	122 ± 31	130 ± 32	132 ± 33	<0.0001
Serum HDL cholesterol (mg/dL)	58 ± 15	55 ± 13	52 ± 13	<0.0001
Lipid-modifying agent use (%)	6.0	8.8	9.9	0.03
Dyslipidemia (%)	37.0	52.2	52.5	<0.0001
eGFR (mi/min/1.73m^2^)	82.9 ± 14.4	79.6 ± 14.1	79.0 ± 13.1	<0.0001

Data are presented as the mean values (standard deviation), percentages, or median (interquartile range)

*3%ODI* 3% oxygen desaturation index, *BMI* body mass index, *HbA1c* hemoglobin A1c, *LDL* low density lipoprotein, *HDL* high density lipoprotein, *eGFR* estimated Glomerular Filtration Rate

Table 2 shows adjusted blood pressure values and the adjusted odds ratios for hypertension according to 3%ODI levels. The age- and sex-adjusted blood pressure values and odds ratio for hypertension increased significantly with higher 3%ODI levels (*p* for trend < 0.0001). These associations remained significant after adjustment for age, sex, current alcohol drinking, current smoking, regular exercise, heart rate, HbA1c, use of glucose-lowering agents, serum LDL cholesterol, serum HDL cholesterol, and eGFR (Table 2). The results remained the same when weekly ethanol consumption and Brinkman index were adjusted rather than current alcohol drinking and current smoking (data not shown). Multi-nominal logistic regression for prehypertension and hypertension showed that the higher the 3%ODI levels, the higher the risks of higher blood pressure categories, which may demonstrate a dose-response relationship (Supplementary Table 1). However, further adjustment for obesity status attenuated the associations (Table 2). Moreover, further adjustment for BMI also attenuated the associations (data not shown).

**Table 2:** Adjusted blood pressure values and the odds ratio for hypertension according to 3%ODI levels in all subjects

	No. of subjects	Model 1	Model 2	Model 3
Systolic blood pressure				
0 ≤ 3%ODI < 5	1804	118.9 ± 1.6	119.2 ± 4.0	119.4 ± 4.0
5 ≤ 3%ODI < 15	627	121.5 ± 1.7	121.1 ± 4.1	120.6 ± 4.0
15 ≤ 3%ODI	101	123.4 ± 2.0	122.0 ± 4.2	120.8 ± 4.2
		*p* for trend < 0.0001	*p* for trend < 0.0001	p for trend<0.0001
Diastolic blood pressure				
0 ≤ 3%ODI < 5	1804	76.2 ± 1.3	76.4 ± 3.1	76.6 ± 3.0
5 ≤ 3%ODI < 15	627	78.2 ± 1.3	77.9 ± 3.1	77.5 ± 3.1
15 ≤ 3%ODI	101	80.0 ± 1.6	79.0 ± 3.2	78.0 ± 3.2
		*p* for trend < 0.0001	*p* for trend < 0.0001	p for trend<0.0001
Odds ratio for hypertension				
0 ≤ 3%ODI < 5	1804	1.00 (Reference)	1.00 (Reference)	1.00 (Reference)
5 ≤ 3%ODI < 15	627	1.50 (1.19–1.88)	1.39 (1.10–1.76)	1.23 (0.96–1.56)
15 ≤ 3%ODI	101	2.18 (1.39–3.42)	1.77 (1.11–2.84)	1.41 (0.88–2.28)
		*p* for trend < 0.0001	*p* for trend = 0.004	*p* for trend = 0.14

Data are presented as the adjusted mean values (standard error) or odds ratio (95% CI)

Model 1: Adjusted for age and sex

Model 2: Adjusted for age, sex, current alcohol drinking, current smoking, regular exercise, heart rate, HbA1c, use of glucose-lowering agents, serum LDL cholesterol, serum HDL cholesterol, and eGFR

Model 3: Adjusted for age, sex, current alcohol drinking, current smoking, regular exercise, heart rate, HbA1c, use of glucose-lowering agents, serum LDL cholesterol, serum HDL cholesterol, eGFR, and obesity status

*3%ODI* 3% oxygen desaturation index, *BMI* body mass index, *95%CI* 95% confidence interval

Hence, we performed the stratified analysis by obesity status (Table 3). The blood pressure values and the odds ratio for hypertension increased significantly with higher 3%ODI only for non-obese individuals, with significant interaction. In a sensitivity analysis, when participants were divided into four groups based on the cutoff value of 3%ODI (3%ODI < 5, 5–10, 10–15, and 15 or more), the similarly significant associations were observed (Supplementary Table 2). We also performed a subgroup analysis of age, however, there was no significant difference in the association between higher 3%ODI and hypertension when the findings were stratified by age (Supplementary Table 3).

**Table 3:** Adjusted blood pressure values and the odds ratios for hypertension according to 3%ODI levels, stratified analysis by obesity status

	No. of subjects	Model 1	Model 2
Systolic blood pressure			
BMI < 25 kg/m^2^			
0 ≤ 3%ODI < 5	1417	117.6 ± 1.9	117.8 ± 5.2
5 ≤ 3%ODI < 15	358	119.2 ± 2.0	118.8 ± 5.2
15 ≤ 3%ODI	37	121.5 ± 2.7	120.3 ± 5.6
		*p* for trend < 0.0001	*p* for trend < 0.0001
BMI ≥ 25 kg/m^2^			
0 ≤ 3%ODI < 5	387	123.3 ± 3.3	123.4 ± 6.8
5 ≤ 3%ODI < 15	269	124.9 ± 3.4	124.9 ± 6.8
15 ≤ 3%ODI	64	124.9 ± 3.7	124.3 ± 7.0
		*p* for trend<0.0001	*p* for trend < 0.0001
Diastolic blood pressure			
BMI < 25 kg/m^2^			
0 ≤ 3%ODI < 5	1417	75.1 ± 1.4	75.2 ± 4.0
5 ≤ 3%ODI < 15	358	76.3 ± 1.5	76.1 ± 4.0
15 ≤ 3%ODI	37	77.5 ± 2.0	76.8 ± 4.3
		*p* for trend < 0.0001	*p* for trend < 0.0001
BMI ≥ 25 kg/m^2^			
0 ≤ 3%ODI < 5	387	80.0 ± 2.5	80.2 ± 5.3
5 ≤ 3%ODI < 15	269	81.2 ± 2.6	81.0 ± 5.3
15 ≤ 3%ODI	64	82.0 ± 2.8	81.5 ± 5.4
		*p* for trend < 0.0001	*p* for trend < 0.0001
Odds ratio for hypertension			
BMI < 25 kg/m^2^			
0 ≤ 3%ODI < 5	1417	1.00 (Reference)	1.00 (Reference)
5 ≤ 3%ODI < 15	358	1.56 (1.15–2.12)	1.47 (1.07–2.02)
15 ≤ 3%ODI	37	2.95 (1.426–6.11)	2.40 (1.13–5.10)
		*p* for trend < 0.001	*p* for trend = 0.008
BMI ≥ 25 kg/m^2^			
0 ≤ 3%ODI < 5	387	1.00 (Reference)	1.00 (Reference)
5 ≤ 3%ODI < 15	269	0.96 (0.67–1.36)	0.95 (0.66–1.37)
15 ≤ 3%ODI	64	1.05 (0.58–1.09)	1.01 (0.54–1.88)
		*p* for trend = 0.94	*p* for trend = 0.95
		*p* for interaction = 0.015	*p* for interaction = 0.03

Data are presented as the adjusted mean values (standard error) or odds ratio (95% confidence interval)

Model 1: Adjusted for age and sex

Model 2: Adjusted for age, sex, current alcohol drinking, current smoking, regular exercise, heart rate, HbA1c, use of glucose-lowering agents, serum LDL cholesterol, serum HDL cholesterol, and eGFR

*3%ODI* 3% oxygen desaturation index, *BMI* body mass index

## Discussion

In this cross-sectional study of an occupational Japanese population, 29% of subjects were suspected to have SRBD, and increased 3%ODI was significantly associated with an increased risk for having hypertension. However, the association was attenuated after adjustment for obesity status. Noteworthy, the influence of increased 3%ODI on having hypertension was more evident for individuals without obesity compared with those of obesity (*p* for interaction = 0.03). These findings suggest that the presence of SRBD should be kept in mind even in non-obese individuals.

One previous retrospective study investigated the association between 3%ODI and blood pressure in Japanese truck drivers [9]. In that study, the odds ratio for having hypertension was significantly higher only when 3%ODI was 15 or higher. When stratified by obesity status (BMI 25 kg/m^2^ or higher), the similar association was observed only in the obese group, without significant interaction. Although the results differ from those of our study, the different definition of hypertension could be one reason for the difference in results. That is, because hypertension was defined as 160/95 mmHg or higher in the previous study, moderate or severe SRBD could be associated with moderate or severe hypertension in that analysis.

Other previous studies that defined hypertension as 140/90 mmHg or higher reported that the odds ratio for hypertension significantly increased according to increased 3%ODI, similar to the present study [2, 3, 5–8]. In contrast, to the best of our knowledge, there has been no stratified analysis by obesity status, with the exception of the study mentioned above [9]. While obesity has been shown to be associated with hypertension and this association was greater in the younger generation [10], in the present study, significant association was observed between higher 3%ODI and higher blood pressure after adjusting for age. Therefore, SRBD may be significantly associated with hypertension regardless of age.

The results of this study could be clinically important because they suggest that SRBD may be present even in non-obese individuals, and that SRBD may be associated with hypertension with a dose-response relationship. Japanese people have small jaws, suggesting the need to watch for the presence of SRBD even in non-obese Japanese individuals. Epidemiological studies have reported that not only hypertension but also high-normal blood pressure (130–139/85–89 mmHg) is associated with risk of developing cardiovascular diseases, especially stroke [22], so better blood pressure control is important even in mild cases of hypertension. Clinically, for example, we should keep in mind that nocturnal hypertension is common in SRBD [23], which would emphasize the importance of home blood pressure monitoring in order to detect clinical findings suggestive of nocturnal hypertension (early morning hypertension in home blood pressure monitoring).

In contrast, the presence and severity of SRBD was not significantly associated with hypertension in obese individuals in the present study. Many previous studies have reported that obesity itself is a risk factor for developing hypertension, which may mask the influence of higher 3%ODI on hypertension. And the effect of obesity on hypertension has been reported to be greater in younger people [10], suggesting the importance of obesity prevention in young generation. In addition, the mechanisms by which SRBD could be associated with hypertension include not only sympathetic nervous system hyperactivity [24], but also increased insulin resistance [25], activation of the Renin-Angiotensin-Aldosterone (RAA) system [26–28], activation of mineralocorticoid receptors [29], oxidative stress [30], and chronic inflammation [31, 32]. These mechanisms overlap with those by which obesity is associated with hypertension, and thus the impact of SRBD on hypertension may not be significant in obese individuals.

The study had several strengths. First, the study enrolled a large occupational population. Second, unlike studies of general populations with a relatively large percentage of elderly, this study included young and middle-aged participants, most of whom were male, and thus was able to examine the association between SRBD and hypertension in young and middle-aged males, which is a problem in Japan. Third, our results were based on reliable data with few missing values, since the participants were required to undergo regular health examinations and simplified PSG.

However, several limitations of this study should be noted. First, selection bias may have occurred because we recruited study participants from among train and bus drivers of a company based in Japan. In addition, the generalizability of the present findings to populations with different genetic backgrounds and lifestyles may have been limited. Second, simplified PSG was used to evaluate SRBD. Full PSG comprehensively assesses biological activities through a variety of measures over the course of a night: electroencephalography, electrocardiography, electromyography, and respiratory curve analysis, along with measurements of snoring, eye movements and oxygen saturation. In contrast, the simplified PSG calculates the 3% oxygen desaturation index (3%ODI: events/h), which is the number of the times when SpO2 decreases by 3% or more per hour. 3%ODI is well known to correlate with AHI and is often used as a screening test [16–19]. However, it does not record sleep. Therefore, the apnea/hypopnea per hour is calculated using the total recording time as the denominator, not the total sleep time. As a result, the AHI may have been underestimated. The simplified PSG has been reported to have an overall sensitivity of 73% when compared to that of the full PSG, but a sensitivity of 52% for AHI < 30, suggesting that the frequency of mild/moderate SRBD may have been underestimated [18]. On the other hand, this does not affect the results of the dose-response relationship for the association between elevated 3%ODI and hypertension. Third, clinical symptoms were not investigated. For occupational safety reasons, it is unlikely that any of the participants would have daytime symptoms (excessive daytime sleepiness, headache, fatigue, and impaired concentration), but it is possible that some would have nighttime symptoms (subjective symptoms: mid-afternoon awakening and nocturia; other symptoms: apnea and snoring). In the present study, however, we defined SRBD based on objective indices regardless of clinical symptoms, following the ICSD-3 definition. Fourth, the impact of the class of antihypertensive agents on the severity of SRBD was not investigated. RAA system inhibitors and diuretics have been reported to improve AHI on SRBD patients [33]; since lack of data prevented that analysis here, those antihypertensive agents might have affected 3%ODI in the present study. Finally, since this was only a cross-sectional study, the causal relationship between 3%ODI elevation and blood pressure elevation remains unclear.

### Perspective of Asia

Asians have been reported to have smaller jaws than other ethnic groups [34]. Further studies, including non-obese Asian individuals are needed to confirm the causal relationship between 3%ODI elevation and blood pressure elevation.

## Conclusions

Higher 3%ODI was significantly associated with higher prevalence of hypertension, especially in non-obese participants, in this Japanese cross-sectional study. Our results suggest the need to be mindful of the potential presence of SRBD even in non-obese individuals.

### Supplementary information

Supplementary Table 1

Supplementary Table 2

Supplementary Table 3
