# Supplementary material for: Sleep-related breathing disorder in a Japanese occupational population and its association with hypertension—stratified analysis by obesity status
Source: Hypertens Res. 2024 Mar 4;47(6):1470–8. doi: 10.1038/s41440-024-01612-y (PMC11150150; doi:10.1038/s41440-024-01612-y)
Supplement: Supplementary file 1 — Supplementary Table 1 [file 41440_2024_1612_MOESM1_ESM.docx]

Supplementary table 1. Multi-nominal logistic analysis according to 3%ODI levels influencing prehypertension and hypertension.

|  |  |  |  | Prehypertension vs. normal BP | | |  | Hypertension vs. normal BP | | |
| --- | --- | --- | --- | --- | --- | --- | --- | --- | --- | --- |
| All subjects |  | No. of subjects |  | Adjusted odds ratio |  | p value |  | Adjusted odds ratio |  | p value |
| Model 1 |  |  |  |  |  |  |  |  |  |  |
| 0≤3%ODI<5 |  | 1804 |  | 1.00 (Reference) |  |  |  | 1.00 (Reference) |  |  |
| 5≤3%ODI<15 |  | 627 |  | 1.34 (1.08-1.66) |  | 0.009 |  | 1.72 (1.34-2.21) |  | <0.0001 |
| 15≤3%ODI |  | 101 |  | 2.32 (1.39-3.85) |  | 0.001 |  | 3.41 (1.97-5.90) |  | <0.0001 |
|  |  |  |  | p for trend<0.0001 | | |  | p for trend<0.0001 | | |
| Model 2 |  |  |  |  |  |  |  |  |  |  |
| 0≤3%ODI<5 |  | 1804 |  | 1.00 (Reference) |  |  |  | 1.00 (Reference) |  |  |
| 5≤3%ODI<15 |  | 627 |  | 1.23 (0.99-1.53) |  | 0.07 |  | 1.53 (1.18-1.99) |  | 0.001 |
| 15≤3%ODI |  | 101 |  | 1.93 (1.15-3.24) |  | 0.01 |  | 2.57 (1.46-4.55) |  | 0.001 |
|  |  |  |  | p for trend=0.004 | | |  | p for trend<0.0001 | | |
| Model 3 |  |  |  |  |  |  |  |  |  |  |
| 0≤3%ODI<5 |  | 1804 |  | 1.00 (Reference) |  |  |  | 1.00 (Reference) |  |  |
| 5≤3%ODI<15 |  | 627 |  | 1.06 (0.85-1.34) |  | 0.59 |  | 1.27 (0.97-1.66) |  | 0.09 |
| 15≤3%ODI |  | 101 |  | 1.45 (0.85-2.45) |  | 0.17 |  | 1.78 (0.99-3.19) |  | 0.05 |
|  |  |  |  | p for trend=0.23 | | |  | p for trend=0.02 | | |

Abbreviations: 3%ODI, 3% oxygen desaturation index; BMI, body mass index; BP, blood pressure; 95%CI, 95% confidence interval

Data are presented as the adjusted odds ratio (95% confidence interval).

Outcome variables were prehypertension and hypertension.

Model 1: Adjusted for age and sex.

Model 2: Adjusted for age, sex, current alcohol drinking, current smoking, regular exercise, heart rate, HbA1c, use of glucose-lowering agents, serum LDL cholesterol, serum HDL cholesterol, and eGFR

Model 3: Adjusted for age, sex, current alcohol drinking, current smoking, regular exercise, heart rate, HbA1c, use of glucose-lowering agents, serum LDL cholesterol, serum HDL cholesterol, eGFR, and obesity status
