# Supplementary material for: Sleep-related breathing disorder in a Japanese occupational population and its association with hypertension—stratified analysis by obesity status
Source: Hypertens Res. 2024 Mar 4;47(6):1470–8. doi: 10.1038/s41440-024-01612-y (PMC11150150; doi:10.1038/s41440-024-01612-y)
Supplement: Supplementary file 2 — Supplementary Table 2 [file 41440_2024_1612_MOESM2_ESM.docx]

Supplementary Table 2.

Adjusted blood pressure values and the odds ratio for hypertension according to four 3%ODI levels, stratified analysis by obesity status

|  |  | No. of subjects |  | Model 1 |  | Model 2 |
| --- | --- | --- | --- | --- | --- | --- |
| Systolic blood pressure |  |  |  |  |  |  |
| BMI<25 kg/m^2^ |  |  |  |  |  |  |
| 0≤3%ODI<5 |  | 1417 |  | 117.6±1.9 |  | 117.8±5.2 |
| 5≤3%ODI<10 |  | 300 |  | 119.2±2.0 |  | 118.9±5.3 |
| 10≤3%ODI<15 |  | 58 |  | 119.4±2.4 |  | 118.5±5.4 |
| 15≤3%ODI |  | 37 |  | 121.5±2.7 |  | 120.3±5.6 |
|  |  |  |  | p for trend<0.0001 |  | p for trend<0.0001 |
| BMI≥25 kg/m^2^ |  |  |  |  |  |  |
| 0≤3%ODI<5 |  | 387 |  | 123.3±3.3 |  | 123.4±6.8 |
| 5≤3%ODI<10 |  | 198 |  | 124.4±3.4 |  | 124.5±6.9 |
| 10≤3%ODI<15 |  | 71 |  | 126.5±3.5 |  | 126.2±6.9 |
| 15≤3%ODI |  | 64 |  | 124.9±3.7 |  | 124.3±7.0 |
|  |  |  |  | p for trend<0.0001 |  | p for trend<0.0001 |
| Diastolic blood pressure |  |  |  |  |  |  |
| BMI<25 kg/m^2^ |  |  |  |  |  |  |
| 0≤3%ODI<5 |  | 1417 |  | 75.1±1.4 |  | 75.2±4.0 |
| 5≤3%ODI<10 |  | 300 |  | 76.1±1.5 |  | 76.0±4.0 |
| 10≤3%ODI<15 |  | 58 |  | 77.4±1.8 |  | 76.8±4.2 |
| 15≤3%ODI |  | 37 |  | 77.5±2.0 |  | 76.8±4.3 |
|  |  |  |  | p for trend<0.0001 |  | p for trend<0.0001 |
| BMI≥25 kg/m^2^ |  |  |  |  |  |  |
| 0≤3%ODI<5 |  | 387 |  | 80.0±2.5 |  | 80.2±5.2 |
| 5≤3%ODI<10 |  | 198 |  | 80.9±2.6 |  | 80.8±5.2 |
| 10≤3%ODI<15 |  | 71 |  | 81.8±2.7 |  | 81.7±5.3 |
| 15≤3%ODI |  | 64 |  | 82.0±2.8 |  | 81.6±5.4 |
|  |  |  |  | p for trend<0.0001 |  | p for trend<0.0001 |
| Odds ratio for hypertension |  |  |  |  |  |  |
| BMI<25 kg/m^2^ |  |  |  |  |  |  |
| 0≤3%ODI<5 |  | 1417 |  | 1.00 (Reference) |  | 1.00 (Reference) |
| 5≤3%ODI<10 |  | 300 |  | 1.52 (1.09-2.12) |  | 1.46 (1.04-2.06) |
| 10≤3%ODI<15 |  | 58 |  | 1.77 (0.94-3.35) |  | 1.52 (0.77-2.96) |
| 15≤3%ODI |  | 37 |  | 2.95 (1.426-6.11) |  | 2.40 (1.13-5.10) |
|  |  |  |  | p for trend=0.007 |  | p for trend=0.02 |
| BMI≥25 kg/m^2^ |  |  |  |  |  |  |
| 0≤3%ODI<5 |  | 387 |  | 1.00 (Reference) |  | 1.00 (Reference) |
| 5≤3%ODI<10 |  | 198 |  | 0.76 (0.51-1.13) |  | 0.77 (0.51-1.17) |
| 10≤3%ODI<15 |  | 71 |  | 1.69 (0.99-2.88) |  | 1.56 (0.89-2.72) |
| 15≤3%ODI |  | 64 |  | 1.05 (0.58-1.09) |  | 1.01 (0.54-1.88) |
|  |  |  |  | p for trend=0.06 |  | p for trend=0.16 |
|  |  |  |  | p for interaction=0.01 |  | p for interaction=0.02 |

Abbreviations: 3%ODI, 3% oxygen desaturation index; BMI, body mass index

Data are presented as the adjusted mean values (standard error) or odds ratio (95% confidence interval).

Model 1: Adjusted for age and sex.

Model 2: Adjusted for age, sex, current alcohol drinking, current smoking, regular exercise, heart rate, HbA1c, use of glucose-lowering agents, serum LDL cholesterol, serum HDL cholesterol, and eGFR
