# Supplementary material for: Sleep-related breathing disorder in a Japanese occupational population and its association with hypertension—stratified analysis by obesity status
Source: Hypertens Res. 2024 Mar 4;47(6):1470–8. doi: 10.1038/s41440-024-01612-y (PMC11150150; doi:10.1038/s41440-024-01612-y)
Supplement: Supplementary file 3 — Supplementary Table 3 [file 41440_2024_1612_MOESM3_ESM.docx]

Supplementary Table 3.

Adjusted blood pressure values and the odds ratio for hypertension according to four 3%ODI levels, stratified analysis by age (40 years or more)

|  |  | No. of subjects |  | Model 1 |  | Model 2 |
| --- | --- | --- | --- | --- | --- | --- |
| Systolic blood pressure |  |  |  |  |  |  |
| age<40 years |  |  |  |  |  |  |
| 0≤3%ODI<5 |  | 653 |  | 114.2±2.1 |  | 114.8±7.9 |
| 5≤3%ODI<15 |  | 147 |  | 118.3±2.2 |  | 116.4±8.1 |
| 15≤3%ODI |  | 21 |  | 121.0±3.2 |  | 115.8±8.6 |
|  |  |  |  | p for trend<0.0001 |  | p for trend<0.0001 |
| age 40 years or more |  |  |  |  |  |  |
| 0≤3%ODI<5 |  | 1151 |  | 121.0±1.7 |  | 121.5±4.5 |
| 5≤3%ODI<15 |  | 480 |  | 123.7±1.7 |  | 122.9±4.6 |
| 15≤3%ODI |  | 80 |  | 125.2±2.1 |  | 123.3±4.7 |
|  |  |  |  | p for trend<0.0001 |  | p for trend<0.0001 |
| Diastolic blood pressure |  |  |  |  |  |  |
| age<40 years |  |  |  |  |  |  |
| 0≤3%ODI<5 |  | 653 |  | 71.1±1.8 |  | 71.8±6.6 |
| 5≤3%ODI<15 |  | 147 |  | 75.1±1.9 |  | 72.8±6.7 |
| 15≤3%ODI |  | 21 |  | 77.0±2.8 |  | 71.1±7.2 |
|  |  |  |  | p for trend<0.0001 |  | p for trend<0.0001 |
| age 40 years or more |  |  |  |  |  |  |
| 0≤3%ODI<5 |  | 1151 |  | 78.5±1.2 |  | 78.8±3.3 |
| 5≤3%ODI<15 |  | 480 |  | 80.4±1.2 |  | 79.9±3.3 |
| 15≤3%ODI |  | 80 |  | 82.1±1.5 |  | 80.7±3.4 |
|  |  |  |  | p for trend<0.0001 |  | p for trend<0.0001 |
| Odds ratio for hypertension |  |  |  |  |  |  |
| age<40 years |  |  |  |  |  |  |
| 0≤3%ODI<5 |  | 653 |  | 1.00 (Reference) |  | 1.00 (Reference) |
| 5≤3%ODI<15 |  | 147 |  | 2.38 (1.30-4.33) |  | 1.20 (0.61-2.36) |
| 15≤3%ODI |  | 21 |  | 2.93 (0.82-10.49) |  | 0.80 (0.18-3.59) |
|  |  |  |  | p for trend=0.009 |  | p for trend=0.59 |
| age 40 years or more |  |  |  |  |  |  |
| 0≤3%ODI<5 |  | 1151 |  | 1.00 (Reference) |  | 1.00 (Reference) |
| 5≤3%ODI<15 |  | 480 |  | 1.53 (1.21-1.94) |  | 1.30 (1.00-1.67) |
| 15≤3%ODI |  | 80 |  | 2.18 (1.36-3.49) |  | 1.56 (0.95-2.58) |
|  |  |  |  | p for trend<0.0001 |  | p for trend=0.05 |
|  |  |  |  | p for interaction=0.37 |  | p for interaction=0.63 |

Abbreviations: 3%ODI, 3% oxygen desaturation index

Data are presented as the adjusted mean values (standard error) or odds ratio (95% confidence interval).

Model 1: Adjusted for sex.

Model 2: Adjusted for sex, obesity status, current alcohol drinking, current smoking, regular exercise, heart rate, HbA1c, use of glucose-lowering agents, serum LDL cholesterol, serum HDL cholesterol, and eGFR
